# Best evidence summary of screening and management of stigma in patients with colorectal cancer and stomas

**DOI:** 10.3389/fonc.2026.1841569

**Published:** 2026-06-04

**Authors:** Jiangfeng Dong, Huanhuan Cheng, Xueping Jiao, Lijuan Song, Qiaohong Niu

**Affiliations:** 1 Cancer Hospital Affiliated to Shanxi Medical University, Taiyuan, Shanxi, China; 2 School of Nursing, Shanxi Medical University, Taiyuan, Shanxi, China; 3 Department of Colorectal Surgery, Shanxi Province Cancer Hospital/Shanxi Hospital Affiliated to Cancer Hospital, Chinese Academy of Medical Sciences/Cancer Hospital Affiliated to Shanxi Medical University, Taiyuan, Shanxi, China; 4 Nursing Department, Shanxi Province Cancer Hospital/Shanxi Hospital Affiliated to Cancer Hospital, Chinese Academy of Medical Sciences/Cancer Hospital Affiliated to Shanxi Medical University, Taiyuan, Shanxi, China

**Keywords:** colorectal cancer, evidence summary, evidence-based nursing, stigma, stoma

## Abstract

**Objective:**

To systematically appraise and synthesize the best available evidence on screening and management strategies for stigma among patients with colorectal cancer and stomas, providing evidence-based support for clinical practice.

**Methods:**

This study was conducted and reported in accordance with the PRISMA guidelines where applicable and the evidence summary development process proposed by the Fudan University Center for Evidence-based Nursing. Guided by the “5S” evidence pyramid model, a top-down systematic search was conducted across decision-support systems, guideline websites, professional organization websites, and major databases. The search period spanned from database inception to January 21, 2026. Multiple reviewers independently performed literature screening, critical appraisal, and evidence extraction.

**Results:**

A total of 17 publications were included, comprising two clinical decisions, six clinical practice guidelines, two expert consensuses, three evidence summaries, one systematic review, and three randomized controlled trials. The synthesized evidence generated seven thematic domains: team construction, stigma identification and assessment, educational guidance, stoma and appliance management, self-management, psychosocial support, and follow-up, yielding 32 evidence-based recommendations.

**Conclusion:**

The 32 evidence-based recommendations across seven domains provide clinically meaningful guidance for screening and managing stigma in patients with colorectal cancer and stomas. These recommendations offer an evidence-based framework for improving clinical practice. During implementation, healthcare professionals should critically appraise the applicability of evidence by considering clinical context, individual patient differences, and family and social backgrounds to optimize stigma reduction and psychosocial adaptation.

## 1 Introduction

Colorectal cancer (CRC) is one of the leading malignancies worldwide in terms of both incidence and mortality and has become a major public health concern threatening global health (1) Substantial advances have been achieved in the treatment of colorectal cancer, including surgical intervention, pharmacological therapy, and radiotherapy. However, surgery remains the primary treatment modality in clinical practice, and approximately 5%–30% of patients require stoma creation (2, 3). Although stoma formation can effectively improve the prognosis of patients with colorectal cancer and prolong survival (4), stoma-related challenges (e.g., altered body image, the risk of odor and leakage, and restricted sexual activity) may lead to varying degrees of stigma among patients (5, 6).

The core concept of stigma originates from Goffman’s stigma theory, which conceptualizes stigma as a socially constructed process of “marked difference” whereby negative labeling damages social identity and results in social exclusion (7). This process may subsequently give rise to negative psychological responses, including inferiority, self-denial, and social withdrawal, and encompasses multiple dimensions such as internalized stigma, perceived stigma, and enacted social stigma (8). Among patients with colorectal cancer and stomas, stigmatization is often characterized by the interaction of multiple forms of stigma. For example, negative social feedback may reinforce patients’ internalized stigma, whereas perceived stigma may adversely affect self-perception and social participation. Therefore, although these dimensions are conceptually distinct, they are frequently regarded as a comprehensive psychosocial burden. The Health Stigma and Discrimination Framework developed by Stangl (7) conceptualizes stigma as a dynamic process involving drivers and facilitators, stigma manifestations, and outcomes. This framework highlights stigma as a critical barrier influencing patients’ care-seeking behaviors, disease perception, and treatment adherence.

Previous studies have demonstrated that stigma among patients with colorectal cancer and stomas is generally at a moderate level, with approximately 44% of patients experiencing high levels of stigma (9). The development of this stigma is closely associated with anxiety and depression, levels of social support, and quality of life (10, 11).Stigma not only substantially reduces patients’ acceptance of the stoma and stoma-care self-efficacy, but may also hinder the restoration of social roles and psychosocial adaptation, thereby exerting persistent negative effects on the long-term prognosis and rehabilitation outcomes of these patients (12–14). Therefore, timely screening and management of stigma in this population are of considerable clinical importance.

However, current evidence regarding the screening and management of stigma among patients with colorectal cancer and stomas remains fragmented and generalized, with a lack of systematic synthesis based on evidence-based methodologies, thereby limiting its application in standardized clinical practice. To address this gap, this study adopted the evidence summary development process proposed by the Fudan University Center for Evidence-based Nursing, which is grounded in the methodological framework established by the Joanna Briggs Institute (JBI) Centre for Evidence-Based Health Care. This process includes question formulation, literature retrieval, study selection, methodological quality appraisal, evidence synthesis and grading, and the development of practice recommendations (15, 16). It offers several advantages, including a focused research topic, reliable evidence sources, comprehensive retrieval strategies, concise content presentation, and ease of dissemination.

Based on this framework, the present study aimed to systematically retrieve, critically appraise, and synthesize relevant evidence regarding the screening and management of stigma among patients with colorectal cancer and stomas. Furthermore, this study sought to develop clear, scientifically grounded, and clinically applicable nursing practice recommendations to provide evidence-based guidance for healthcare professionals in the screening and management of stigma in this patient population.

## 2 Methods

This study was conducted and reported in accordance with the PRISMA guidelines where applicable and the evidence summary development process proposed by the Fudan University Center for Evidence-based Nursing to ensure methodological rigor and transparency, thereby enhancing the credibility and reproducibility of the study. The study protocol was registered with the Fudan University Center for Evidence-based Nursing (Registration No. ES20259222).

### 2.1 Construction of evidence-based questions

The review question was formulated as follows: What is the best available evidence for the screening and management of stigma among patients with colorectal cancer and stomas? A structured evidence-based question was developed according to the PIPOST framework (17). P (Population): The target population of the evidence application comprised patients with colorectal cancer and stomas; I (Intervention): The intervention referred to stigma-related screening and management strategies; P (Professional): The applied evidence professional included healthcare workers, patients and their caregivers; O (Outcome): The outcome focused on improvements in patients’ stigma status; S (Setting): The application setting encompassed healthcare institutions, communities and families; T (Type of evidence): The type of evidence included clinical decisions, clinical practice guidelines, expert consensuses, evidence summaries, systematic reviews and original studies.

### 2.2 Search strategy

**Figure 1:**
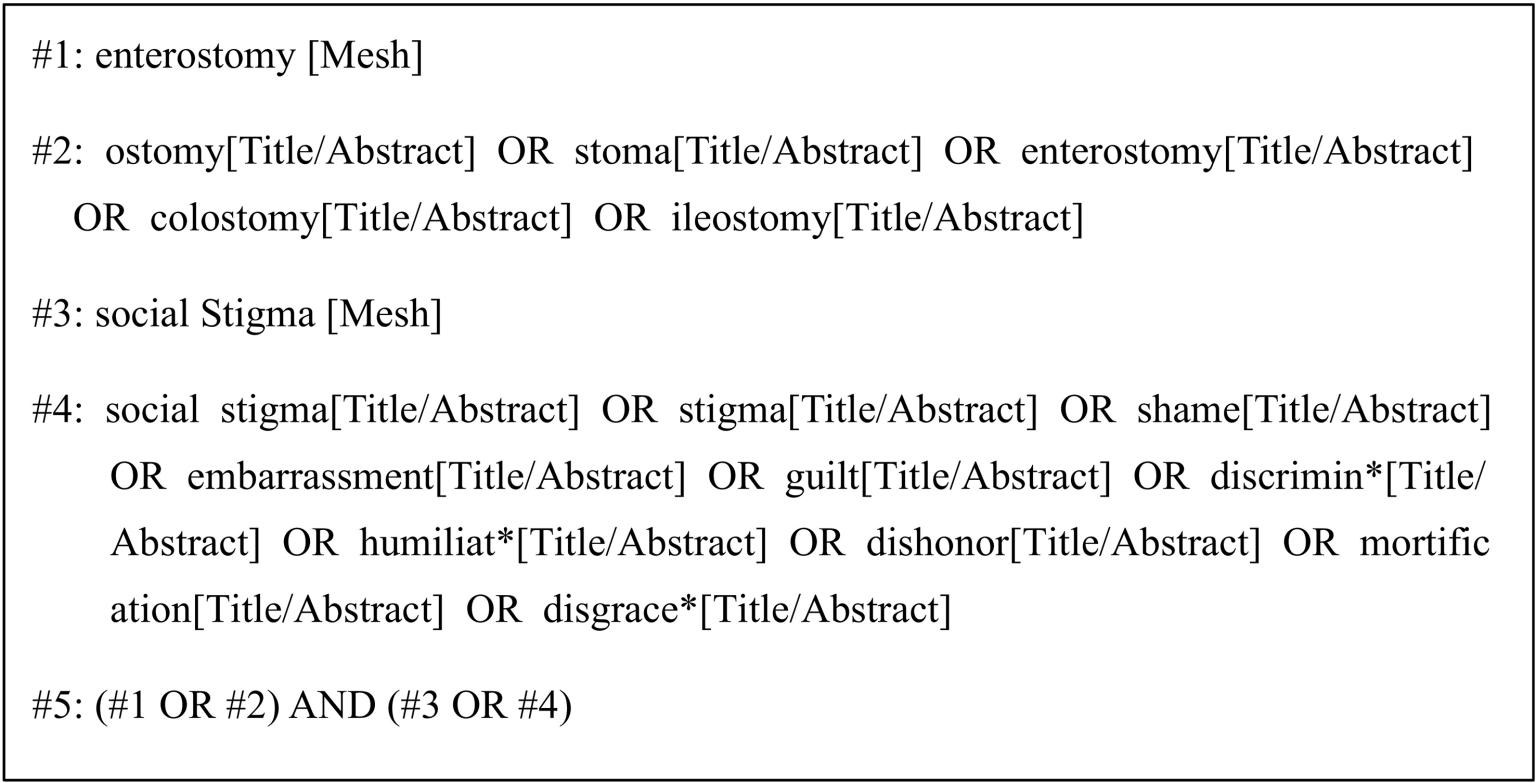
Search strategy for the PubMed.

According to the top-down approach of the 5S evidence pyramid model (18), higher-level evidence resources were prioritized to ensure the provision of reliable guidance for clinical decision support systems. Evidence retrieval and integration were conducted sequentially in descending order of the evidence hierarchy: clinical decisions, evidence summaries, clinical practice guidelines, systematic reviews and original studies. These included UpToDate, BMJ Best Practice, Guidelines International Network (GIN), National Comprehensive Cancer Network (NCCN), Registered Nurses’Association of Ontario (RNAO), National Institute for Health and Care Excellence (NICE), Scottish Intercollegiate Guidelines Network (SIGN), YI Maitong, World Council of Enterostomal Therapists (WCET), Nurses Specialized in Wound,Ostomy and Continence Canada (NSWOCC), United Ostomy Association of Americ a(UOAA), Wound, Ostomy, And Continence Nurses Society (WOCN), American Society of Colon and Rectal Surgeons(ASCRS), Chinese Nursing Association (CNA), Joanna Briggs Institute (JBI), Cochrane Library, CINAHL, Embase, Web of Science, PubMed, China National Knowledge Infrastructure (CNKI), VIP Database, Wanfang Database, China Biology Medicine Database (CBM). A combination of subject terms and free terms was adopted for the search, with “ostomy or stoma or enterostomy or colostomy or ileostomy” and “social stigma or stigma or shame or embarrassment or guilt or discrimin* or humiliat* or dishonor or mortification or disgrace*” as the search terms. The search time limit was from the establishment of the database to January 21, 2026, and some additional articles were manually retrieved. The search strategy used for PubMed is presented in **Figure 1**, and the strategies for other databases were adapted as appropriate.

### 2.3 Inclusion and exclusion criteria for literature

Inclusion criteria: (a) Studies involving patients with colorectal cancer and stomas; (b) Research focusing on the screening and management of stigma; (c) The literature types include clinical decisions, clinical practice guidelines, expert consensuses, evidence summaries, systematic reviews, and original studies, (d) Publications available in either Chinese or English.

Exclusion criteria: (a) Literature with incomplete content information, duplicate publication, or unavailable full texts; (b) Translated, interpreted, or outdated versions of the literature or guidelines; (c) Studies failing to meet the predefined methodological quality evaluation standards.

### 2.4 Evaluation of literature quality

Appropriate literature quality assessment tools were employed tailored to the specific types of literature. The quality of the guidelines was evaluated using the Appraisal of Guidelines for Research and Evaluation II (AGREE II) (19). This tool encompasses 6 domains and 23 specific items, alongside 2 overall assessment items, with each rated on a 7-point Likert scale. The guidelines were classified into 3 levels based on the standardized percentage scores of each domain. Among them, level A (recommended) was defined as having a standardized score percentage of ≥ 60% in all 6 domains, level B (recommended after revision) was defined as having a standardized percentage of ≥ 30% in at least 3 domains and at least 1 domain < 60%, and level C (not recommended) was defined as having a score < 30% in 3 or more domains. Expert consensuses, systematic reviews, and randomized controlled trials were evaluated respectively using the corresponding literature quality assessment tools of the corresponding JBI critical appraisal tools (20–22), Each item was judged using the options of “yes”, “no”, “unclear”, and “not applicable”. Regarding clinical decisions and evidence summaries, the underlying evidence was traced back to its primary source literature, which was then evaluated using the respective quality appraisal tools. The guidelines were independently evaluated by four researchers, while the evaluation of other types of literature was completed by two researchers. In case of disagreement in evaluation opinions, the final judgment was determined through discussion among the members. All the researchers involved in the evaluation of literature quality had completed systematic training in evidence-based nursing methodology.

### 2.5 Summary and classification of evidence

Evidence extraction and synthesis were independently conducted by two researchers who had received systematic training in evidence-based nursing. When the extracted evidence was consistent across studies, evidence with similar content was summarized using clear, concise, and professionally appropriate language. When the evidence was complementary, findings were integrated into a coherent and complete statement based on logical relationships. In cases of conflicting evidence, priority was given to evidence with a higher level of rigor according to evidence-based principles, including studies with more robust methodologies, more comprehensive content, and recently published authoritative literature. The levels of evidence for the included studies were graded from Level 1 to Level 5 according to the 2014 version of the JBI Levels of Evidence and Grades of Recommendation system (23).

## 3 Results

### 3.1 Literature search process and basic characteristics of the included literature

**Figure 2:**
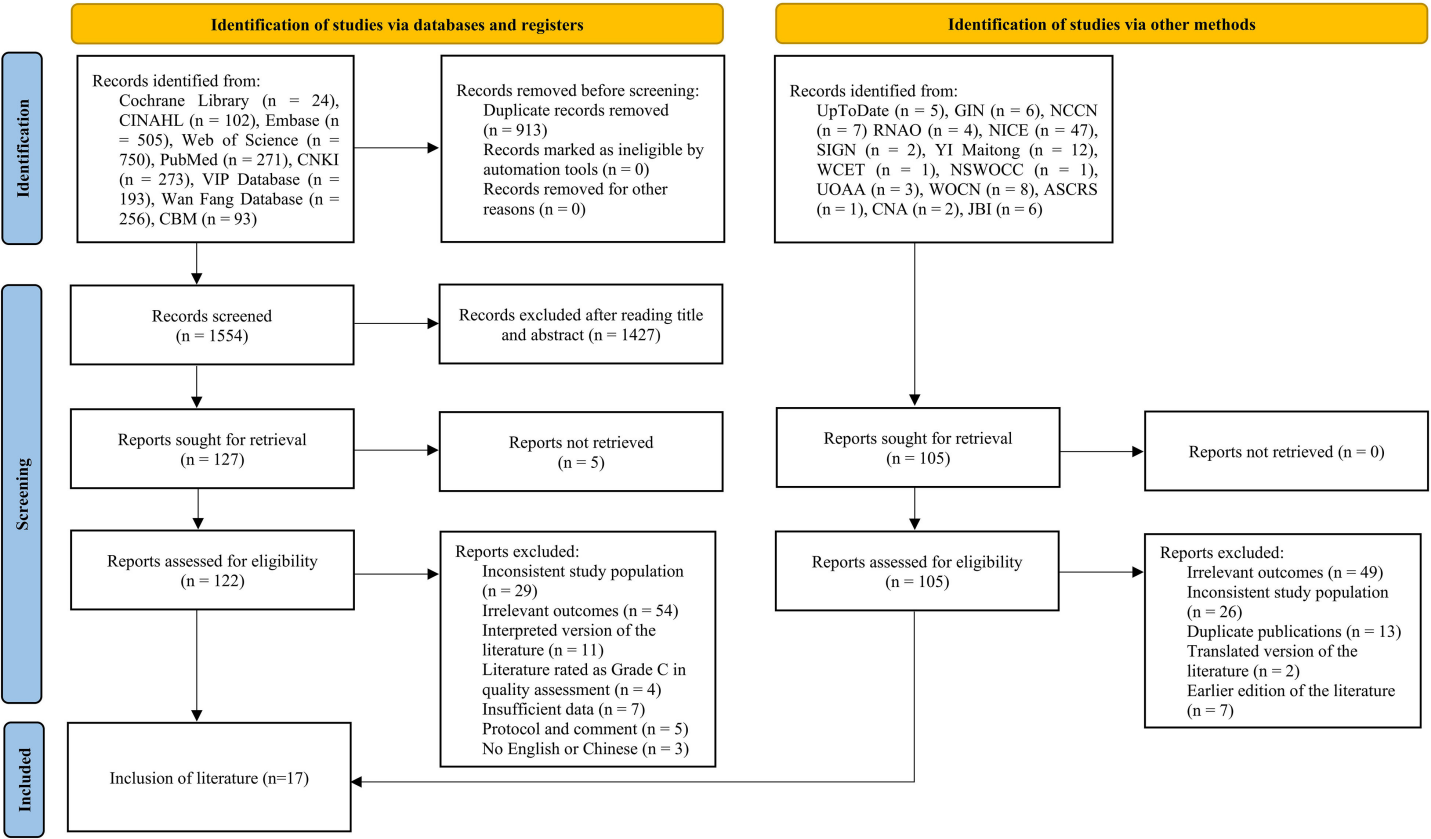
The PRISMA flow diagram.

A total of 2,572 records were initially identified through the comprehensive literature search. After removing duplicate records and conducting screening based on titles, abstracts, and full-text review, 17 studies were ultimately included. This final selection comprised 2 clinical decisions (24, 25), 6 clinical practice guidelines (26–31), 2 expert consensuses (32, 33), 3 evidence summaries (34–36), 1 systematic review (37), and 3 randomized controlled trials (38–40). The literature selection process based on the PRISMA flow diagram is presented in **Figure 2**, and the general characteristics of the included literature are shown in **Table 1**.

**Table 1:** Characteristics of included literature (n=17).

Included literature	Year of publication	Literature sources	Type of literature	Literature themes
Landmann et al. (24)	2021	UpToDate	clinical decision	Ileostomy or colostomy care and complications
Francone et al. (25)	2021	UpToDate	clinical decision	Overview of surgical ostomy for fecal diversion
Wound, Ostomy, and Continence Nursing Specialty Group of Chinese Nursing Association et al. (26)	2025	Wan Fang	Guideline	Guideline for adult enterostomy care
RNAO (27)	2019	RNAO	Guideline	Supporting Adults Who Anticipate or Live with an Ostomy: Second Edition
Chabal et al. (28)	2020	WCET	Guideline	Practice Implications from the WCET^®^ International Ostomy Guideline 2020
Miller et al. (29)	2017	WOCN	Guideline	Executive Summary: Enhanced Recovery After Surgery: Best Practice Guideline for Care of Patients With a Fecal Diversion
WOCN (30)	2018	WOCN	Guideline	WOCN Society Clinical Guideline: Management of the Adult Patient With a Fecal or Urinary Ostomy-An Executive Summary
Aubert et al. (31)	2024	PubMed	Guideline	Management of adult intestinal stomas: The 2023 French guidelines
Ostomy Professional Committee, Chinese Society of Coloproctology, Chinese Medical Doctor Association et al. (32)	2022	Wan Fang	Expert Consensus	Chinese expert consensus on protective ostomy for mid⁃low rectal cancer
Colorectal Surgery Group, Society of Surgery, Chinese Medical Association et al. (33)	2025	Wan Fang	Expert Consensus	Expert consensus on permanent ostomy for colorectal cancer
Wu et al. (34)	2025	CNKI	Evidence summary	Screening and management of psychological distress in the patients with ostomy for colorectal cancer
Sivapuram et al. (35)	2022	JBI	Evidence summary	Stoma: Care and Assessment
Magtoto et al. (36)	2023	JBI	Evidence summary	Stoma Care: Post-operative Patient Assessment and Education
Wang et al. (37)	2023	Wan Fang	Systematic review	The influencing factors of stigma in patients with stoma
Qu et al. (38)	2024	CNKI	Randomized controlled trial	Effects of positive psychological intervention in patients with permanent enterostomy
Zheng et al. (39)	2025	Wan Fang	Randomized controlled trial	Effects of happy PERMA intervention on resilience, stigma and quality of life in colostomy patients with colorectal cancer
Li et al. (40)	2024	Wan Fang	Randomized controlled trial	Effect of narrative nursing on improving stigma, stoma adaptation and quality of life in patients with permanent enterostomy

### 3.2 Results of the evaluation of the quality of the included literature

#### 3.2.1 Quality evaluation results of the clinical decisions

A total of two clinical decisions were included (24, 25). The original studies from which the evidence was derived were traced and appraised using the corresponding methodological quality assessment tools developed by the corresponding JBI critical appraisal tools (20–22). Through the review and evaluation process, it was considered that the overall methodological quality of these retrieved studies was high.

#### 3.2.2 Quality evaluation results of clinical practice guidelines

Six clinical practice guidelines were included in this study (26–31), with their quality appraisal results detailed in **Table 2**.

**Table 2:** Quality evaluation results of clinical practice guidelines.

Guidelines	Percentage of standardization in each field of the guideline (%)						The number of fields with a proportion of ≥ 60% (unit)	The number of fields with a proportion of ≥30% (unit)	Level
	Scope and purpose	Stakeholder involvement	Rigor of development	Clarity of presentation	Applicability	Editorial independence			
Wound, Ostomy, and Continence Nursing Specialty Group of Chinese Nursing Association et al. (26)	98.61	84.72	69.79	91.67	76.04	87.50	6	6	A
RNAO et al. (27)	97.22	90.27	88.54	93.05	95.83	91.67	6	6	A
Chabal et al. (28)	86.11	73.61	63.54	79.17	77.08	58.33	5	6	B
Miller et al. (29)	90.28	88.89	70.83	84.72	72.91	56.25	5	6	B
WOCN et al. (30)	95.83	88.89	86.98	87.50	59.36	68.75	5	6	B
Aubert et al (31)	98.61	94.44	88.02	90.28	89.58	85.42	6	6	A

#### 3.2.3 Quality evaluation results of expert consensuses

The methodological quality appraisal results for the two included expert consensuses (32, 33) are shown in **Table 3**.

**Table 3:** Quality evaluation results of expert consensuses.

Expert consensus	Evaluation items					
	①	②	③	④	⑤	⑥
Ostomy Professional Committee, Chinese Society of Coloproctology, Chinese Medical Doctor Association et al. (32)	Yes	Yes	Yes	Yes	Yes	No
Colorectal Surgery Group, Society of Surgery, Chinese Medical Association et al. (33)	Yes	Yes	Yes	Yes	Yes	No

① Is the source of the opinion clearly identified? ② Does the source of opinion have standing in the field of expertise? ③ Are the interests of the relevant population the central focus of the opinion? ④ Does the opinion demonstrate a logically defended argument to support the conclusions drawn? ⑤ Is there reference to the extant literature? ⑥ Is any incongruence with the literature/sources logically defended?

#### 3.2.4 Quality evaluation results of evidence summaries

For the included evidence summaries, the original studies from which the evidence was extracted were traced and appraised using the corresponding JBI critical appraisal tools (20–22). The overall methodological quality of the included literature was high, and all were deemed eligible for inclusion (34–36).

#### 3.2.5 Quality evaluation results of systematic review

The included systematic review (37) demonstrated a relatively comprehensive study design and high methodological quality and was therefore considered eligible for inclusion; the quality appraisal results are presented in **Table 4**.

**Table 4:** Quality evaluation results of systematic review.

Systematic review	Evaluation items										
	①	②	③	④	⑤	⑥	⑦	⑧	⑨	⑩	⑪
Wang et al. (37)	Yes	Yes	Yes	Yes	Yes	Yes	Yes	Yes	No	Yes	Yes

① Is the review question clearly and explicitly stated? ② Were the inclusion criteria appropriate for the review question? ③ Was the search strategy appropriate? ④ Were the sources and resources used to search for studies adequate? ⑤ Were the criteria for appraising studies appropriate? ⑥ Was critical appraisal conducted by two or more reviewers independently? ⑦ Were there methods to minimize errors in data extraction? ⑧ Were the methods used to combine studies appropriate? ⑨ Was the likelihood of publication bias assessed? ⑩ Were recommendations for policy and/or practice supported by the reported data? ⑪ Were the specific directives for new research appropriate?

#### 3.2.6 Quality evaluation results of randomized controlled trials

The three randomized controlled trials exhibited generally high methodological rigor (38–40), with their corresponding assessments summarized in **Table 5**.

**Table 5:** Quality evaluation results of randomized controlled trials.

Randomized controlled trials	Evaluation items												
	①	②	③	④	⑤	⑥	⑦	⑧	⑨	⑩	⑪	⑫	⑬
Qu et al. (38)	Yes	Unclear	Yes	Unclear	Not applicable	Yes	Yes	Yes	Yes	Yes	Yes	Yes	Yes
Zheng et al. (39)	Yes	Unclear	Yes	Unclear	Not applicable	Yes	Yes	Yes	Yes	Yes	Yes	Yes	Yes
Li et al. (40)	Yes	Yes	Yes	Unclear	Not applicable	Unclear	Yes	Yes	Yes	Yes	Yes	Yes	Yes

① Was true randomization used for assignment of participants to treatment groups? ② Was allocation to treatment groups concealed? ③ Were treatment groups similar at the baseline? ④ Were participants blind to treatment assignment? ⑤ Were those delivering the treatment blind to treatment assignment? ⑥ Were treatment groups treated identically other than the intervention of interest? ⑦ Were outcome assessors blind to treatment assignment? ⑧ Were outcomes measured in the same way for treatment groups? ⑨ Were outcomes measured in a reliable way? ⑩ Was follow up complete and if not, were differences between groups in terms of their follow up adequately described and analyzed? ⑪ Were participants analyzed in the groups to which they were randomized? ⑫ Was appropriate statistical analysis used? ⑬ Was the trial design appropriate and any deviations from the standard RCT design (individual randomization, parallel groups) accounted for in the conduct and analysis of the trial?

### 3.3 Summary and description of evidence

The research team conducted an evidence analysis, extraction, and synthesis of the included studies. This resulted in evidence across seven domains: team construction, stigma identification and assessment, educational guidance, stoma and appliance management, self-management, psychosocial support, and follow-up, comprising a total of 32 pieces of evidence. The levels of evidence were classified as follows: 1a-Meta-analysis of homogeneous randomized controlled trials (RCTs), 1b-Systematic review of RCTs and other study designs, 1c-RCT, 2a-Systematic review of quasi-experimental studies, 2b-Systematic review of quasi-experimental and other lower study designs, 4a-Systematic review of descriptive studies, 5b-Expert consensus, 5c-Bench research/single expert opinion. The details are presented in **Table 6**.

**Table 6:** Summary of the evidence.

Evidence items	Content of evidence	Level of evidence
Team Construction	1.It is recommended that a multidisciplinary team comprising stoma care nurses, psychotherapists, and clinicians be formed to provide patients with assessments of stigma, health education, and follow-up care (27, 31, 35).	2a
	2.Encourage patients and their families to jointly develop and implement personalized plans to address stoma-related stigma, thereby improving patients’ acceptance of their stoma and enhancing their self-esteem (28, 35).	5b
Stigma Identification and Assessment	3.A multidisciplinary team conducts a comprehensive assessment of the individual needs, psychological adaptation levels, emotional needs, religious beliefs, and quality of life of patients and their families, identifying potential sources of stigma (27, 28, 32, 33).	2a
	4.Conduct a dynamic assessment of the patient’s sense of stigma, and determine whether there are psychological problems such as self-abandonment, crying, fear of ostomy, refusal to socialize, and issues related to self-identity (27, 34).	4a
	5.It is recommended that stoma-related stigma be incorporated into routine patient assessments to identify high-risk groups prone to experiencing such stigma (with a particular focus on young and middle-aged adults, patients with stoma odor, frequent leakage, poor stoma acceptance, low levels of social support, and low self-efficacy (37).	1a
	6.Assessing the severity of patients’ stigma using the Social Impact Scale (SIS) (37).	1a
Educational Guidance	7.Educational guidance should be presented in a clear and accessible manner (33).	2a
	8.Include both patients and their families in educational guidance programs to enhance their understanding of the disease, correct misconceptions, and better address the psychological distress caused by the stigma associated with the condition (31).	1c
	9.Stoma specialist nurses provide patients with in-depth preoperative counseling and education on disease awareness, stoma surgery, daily living, and self-care, which helps patients understand the changes in their body image in advance and adjust psychologically (24, 25, 27).	1c
	10.On the day of the surgery, comprehensive nursing guidance including assessment of the stoma and the surrounding skin, management of gas and odor, self-management of the stoma (such as changing the ostomy bag process), identification of potential complications, etc. is provided, and this continues until the patient masters the self-care skills. This helps patients enhance their sense of control and build confidence, and reduces the experience of shame (27, 30, 36).	1c
Stoma and Appliance Management	11. Prior to surgery, stoma specialist nurse or physician will conduct a comprehensive assessment of factors such as changes in body position (lying down, sitting, standing, bending), the patient’s occupation, lifestyle, and religious and cultural background to identify at least two potential stoma sites. This approach aims to reduce the risk of complications and minimize feelings of shame from the outset (23, 26, 28, 32).	1a
	12.The stoma is typically located within the rectus abdominis muscle. For obese patients with a body mass index (BMI) of 30 kg/m² or higher, it should be positioned at the highest point of the abdominal wall to facilitate easy visual inspection and palpation, thereby enhancing the patient’s sense of self-management and improving stoma acceptance (26, 29).	1b
	13.When selecting a stoma appliance, taking into account factors such as the type, shape, and location of the stoma, abdominal contour, and lifestyle can help reduce the risk of developing a sense of shame (30).	1c
	14.Choose a stoma appliance that is comfortable, odor-resistant, safe, and provides a good seal. If necessary, use supplementary products (such as anti-leak ointment, adhesive rings, or skin protectants) to enhance the seal, reduce odors and leakage, and protect the skin around the stoma, thereby preventing or alleviating feelings of shame (26, 28, 30, 35).	1c
	15.The stoma base should be trimmed to minimize skin exposure while accommodating the size and shape of the stoma, extending approximately 1–2 mm beyond the base of the stoma (26).	1c
	16.It is recommended to change the stoma baseplate every 3 to 7 days and the stoma pouch 1 to 2 times a week. Empty the pouch promptly when it is 1/3 to 1/2 full to prevent it from becoming too heavy, which can lead to leakage and odor, thereby reducing feelings of embarrassment (24, 26).	1b
Self-Management	17.For individuals with high-output ileostomies, adjusting their diet (such as consuming high-sodium, high-fat foods and complex carbohydrates, and reducing the intake of hypertonic and hypotonic fluids) can help alleviate the stigma caused by uncontrolled bowel movements (24, 30, 31).	1c
	18.Ensure adequate intake of dietary fiber and fluids to prevent bowel irregularities (24).	1c
	19.Try to avoid smoking, drinking carbonated beverages, chewing gum, and irregular meal times. Also, reduce your intake of fermentable, gas-producing foods such as soybeans, celery, and oats to prevent the stigma caused by excessive gas (24, 26).	5c
	20.When emptying the storage bag, you can use deodorants or fragrance sprays to reduce the unpleasant smell (24).	5b
	21.During sexual activities, emptying or replacing the ostomy bag in advance, and using special underwear/waistbands to conceal and secure it can enhance one’s confidence and the sense of security during intimate interactions (24).	2b
	22.Patients are advised to engage in moderate physical exercise and use an abdominal support belt to secure the stoma appliance (24, 34).	5b
	23. Practice appropriate progressive muscle relaxation exercises to relax your body, promote mental relaxation, and alleviate feelings of stigma (35).	5b
Psychosocial Support	24.Guide patients to identify positive events in their daily lives and reflect on their emotional state, with the aim of fostering positive psychological coping mechanisms to address feelings of stigma associated with their stoma (38).	1c
	25.Encourage patients to express their experiences of stigma through specific events or experiences, thereby making their feelings of stigma more concrete and precise (40).	1c
	26.Actively discuss the current issues surrounding the stigma associated with the illness with patients and their families, acknowledge the positive coping behaviors exhibited in the face of these challenges, and boost patients’ confidence in their ability to cope (38, 39).	1c
	27.Guide patients to identify positive experiences that bring a sense of accomplishment, encourage them to leverage their strengths to achieve personalized care goals, and reduce stigma by fostering psychological resilience (39).	1c
	28.Encouraging patients to actively share their experiences to help others can effectively alleviate negative emotions, help them realize their self-worth, and enhance their sense of fulfillment (38–40).	1c
	29.It is recommended that family members participate in the patient’s care and management to provide emotional support, enhance the patient’s social support network, and reduce feelings of stigma (27, 29, 36).	5b
	30.Encourage patients to participate in appropriate social support activities (such as ostomy volunteer programs, interest groups, and ostomy support groups) based on their individual characteristics, to help them accept their body image, strengthen their social support network, and build their confidence in coping with the stigma associated with their condition (33, 34).	1c
Follow-up	31.Standardized follow-up should begin immediately after surgery and continue until the patient is discharged (31).	1c
	32.Follow-up care is led by stoma care nurses, who incorporate mental health education into the follow-up process to help patients adapt to their stoma and alleviate their psychological distress (34, 35).	1b

## 4 Discussion

### 4.1 Team development and collaborative management provide a foundation for screening and managing stigma among patients with colorectal cancer and stomas

The presence of a stoma often exposes patients to multiple physical and psychosocial adaptation challenges, including changes in bowel habits, risks of odor and leakage, diminished family roles, and social withdrawal. In an attempt to mitigate the emotional distress elicited by these challenges, patients frequently adopt avoidance and withdrawal behaviors. This withdrawal reinforces negative self-evaluations, thereby acting as a catalyst for stigmatization (41, 42). Previous studies have shown that the occurrence and development of disease stigma are not only an isolated psychological experience but also closely related to public disease cognition, interpersonal interaction, and social environmental factors (10). Therefore, interventions relying on a single discipline alone are insufficient to comprehensively address the development and persistence of stigma, highlighting the urgent need for an integrated biopsychosocial multidisciplinary management model.

Furthermore, the presence of a stoma often increases patients’ dependence on family members, underscoring the critical role of family involvement in stoma management. Family-centered care planning and shared decision-making, may help reduce stigma levels among patients with colorectal cancer and stomas, improve family members’ acceptance of the stoma, and further enhance patients’ trust in healthcare professionals and adherence to care (43). Therefore, establishing a multidisciplinary management team that includes healthcare professionals, psychotherapists, and family members may facilitate patients’ adaptation to life with a stoma across physiological, psychological, and social dimensions, thereby indirectly alleviating stigma by addressing multiple contributing factors.

Notably, although the development of multidisciplinary management teams has been recommended as a general stoma care strategy in high-level evidence sources, including clinical practice guidelines, evidence summaries, and expert consensuses (27, 32, 35), direct evidence supporting its effectiveness in reducing stigma remains limited. Based on the synthesized evidence, it is recommended that multidisciplinary team construction should clearly define professional roles and collaborative pathways (27, 31, 35). The stoma specialist nurse should take a leading role, integrating standardized nursing care plans with psychological interventions delivered by mental health professionals and emotional support from family members and other collaborative paths to provide a systematic, continuous, and standardized support network for patients. By transcending the inherent limitations of siloed care models, this paradigm shift offers a systematic, continuous, and standardized support network, establishing a robust framework that places equal and paramount emphasis on the physiological, psychological, and social dimensions of patient care.

### 4.2 Comprehensive and dynamic assessment is an important prerequisite for identifying stigma

Early psychological screening and assessment for cancer patients can not only help healthcare providers accurately grasp the psychological state of the patients, but also enable timely intervention measures (44). However, in current clinical practice, the identification of stigma among patients with colorectal cancer and stomas mainly relies on the patients’ self-description or the subjective feelings of healthcare providers, making it difficult to promptly detect latent or early-stage stigma. This may delay optimal intervention windows and subsequently impair patients’ psychosocial adaptation and overall quality of life. Currently, many studies focus on the influencing factors and intervention measures of stigma in patients with colorectal cancer and stomas, whereas research on stigma assessment and screening remains insufficient (45). Therefore, introducing systematic and standardized psychological screening tools such as the Social Impact Scale (SIS) into clinical practice is of great significance for dynamic assessment and the discovery of potential stigma. Such tools may facilitate the timely recognition of high-risk populations and provide a basis for subsequent individualized interventions.

At the same time, psychological screening should not be regarded as a one-time assessment, but rather as an integral component throughout the entire continuum of patient management. For patients with colorectal cancer and stomas, the challenges they face vary significantly across different stages. In the early postoperative period, they mainly focus on the fear caused by changes in body image and inability to control defecation. After discharge and reintegration into society, patients are more susceptible to factors such as social anxiety and diminished social roles, which can trigger or exacerbate stigmatization (46, 47). Therefore, a phased, comprehensive assessment system should be implemented to conduct dynamic monitoring, aligned with key time points in the psychological adaptation of patients. Furthermore, the level of attention healthcare providers give to screening for stigmatization is equally critical. In clinical practice, psychological problem screening and identification are often not given due attention, resulting in inadequate implementation of stigmatization screening. Therefore, through standardized training and the establishment of institutional frameworks, stigmatization screening should be incorporated into routine assessments to ensure the continuity and standardization of screening at the institutional level. Although the Social Impact Scale has been supported by direct evidence, including systematic reviews and randomized controlled trials in patients with colorectal cancer and stomas (37–40), certain items may fail to fully capture stoma-specific experiences, thereby lacking population specificity. Moreover, given its heavy reliance on subjective self-assessment, cultural differences can significantly skew patient responses, potentially driving considerable cross-regional variations in reported stigma levels. Currently, SIS-based evaluations rely primarily on cumulative scores, where higher values denote greater stigma. However, the absence of a universally established clinical cutoff value remains a critical limitation, often delaying the identification of high-risk individuals. Therefore, future research should focus on developing culturally adapted and population-specific stigma assessment tools tailored to the characteristics and sociocultural contexts of patients with stomas in different countries. Establishing clinically meaningful threshold values to distinguish varying levels of stigma may further improve the accuracy and effectiveness of stigma screening and assessment among patients with colorectal cancer and stomas, thereby providing a quantitative basis for targeted interventions.

### 4.3 Rich, intuitive, and effective individualized educational guidance is key to promoting patients’ psychological adaptation

The presence of a stoma requires patients to undergo both short-term acceptance and long-term adaptation processes. During this period, a lack of lack relevant knowledge about stoma care may lead to a series of negative behaviors, thereby adversely affecting disease prognosis and psychological adaptation (47). Research shows that due to the widespread implementation of Enhanced Recovery After Surgery (ERAS) protocols, many patients are discharged without adequate preparation, resulting in insufficient patient education regarding stoma care. As a result, patients often demonstrate substantial needs for professional stoma-related information and educational support during the stoma rehabilitation and adaptation stages (46, 48). Therefore, providing educational interventions may gradually alleviate negative psychological responses caused by inadequate stoma knowledge and indirectly reduce stigma among patients with colorectal cancer and stomas.

Meanwhile, family caregivers who lack adequate stoma-related knowledge may also develop negative perceptions and experience courtesy stigma due to their close relationship with the patient (49). Therefore, educational guidance should not only target the patients themselves but also include their family members. Research has found that education led by stoma specialist nurses can reduce stigma levels among patients with colorectal cancer and stomas; however, the content and delivery methods of current health education programs remain largely homogeneous and often lack individualized consideration (48). Such approaches may be insufficient to meet patients’ actual needs, difficult to address the deeper psychological concerns of patients and their families, and may even exacerbate anxiety and worry due to an excessive informational burden or unsuitable teaching methods. Consequently, higher demands are placed on nurses. In addition to strengthening training in diversified educational approaches and content delivery, nurses should enhance their innovative capabilities and establish patient-centered interactive educational models. Educational strategies should fully consider patients’ family circumstances, cultural backgrounds, cognitive levels, and psychological tolerance. Furthermore, individualized education should be integrated with dynamic and interactive teaching approaches, such as gamified education, the teach-back method, and scenario simulations to facilitate the transition from passive knowledge acquisition to active health information seeking among patients (50, 51).

Psychological support is equally indispensable in educational guidance. By embedding emotional communication and cognitive restructuring throughout the entire educational process, and by paying close attention to patients’ verbal and nonverbal cues, nurses can use empathetic communication and positive feedback to help patients confront feelings of shame, avoidance, or denial. This approach enables patients to gradually accept physical changes, rebuild their self-identity, and strengthen both patients’ and their families’ understanding, acceptance, and support regarding stomas.

But, given the limited evidence specifically evaluating the effects of educational intervention strategies on stigma among patients with colorectal cancer and stomas, future studies should conduct high-quality research to further explore how differences in educational content, delivery methods, and intervention frequency influence stigma outcomes in this population.

### 4.4 Precise stoma management and scientific selection of stoma devices are key pathways to control the externality of stigma

During the postoperative adaptation process to the stoma, patients often face issues such as stoma odor, fecal leakage, and skin irritation due to improper selection of stoma appliances and misuse of accessories. These problems not only lead to negative self-perception and stigma but also impede the restoration of daily functioning, occupational reintegration, and the maintenance of interpersonal relationships (12, 52). Therefore, implementing refined stoma management and scientifically selecting stoma devices are imperative for controlling the externality of stigma and blocking its internal evolution. These technical general stoma care measures may indirectly prevent or alleviate stigma by reducing “stigma-triggering factors”.

Taking into account both pathophysiological factors and individual differences, formulating an individualized stoma site selection plan and placing the stoma in a position where the patient can easily see and touch it is not only an effective measure to reduce the risk of postoperative stoma complications and significantly improve self - care ability, but also a prerequisite for the precise selection of stoma devices (53). The selection of stoma devices and accessories should consider not only functional appropriateness but also balance the patient’s need for discretion and quality of life.

Products characterized by lightweight design, enhanced comfort, odor resistance, and transparent inspection windows may help increase patients’ confidence in social and daily-life settings while facilitating self-observation and self-management (26, 35). Such approaches may enhance patients’ sense of control over the stoma and indirectly reduce stigma levels. However, as the stoma heals post-surgery and is influenced by factors such as weight fluctuations and lifestyle changes, stoma morphology and peristomal skin condition will also change accordingly, which may render the original stoma device no longer suitable. Therefore, stoma specialist nurses can utilize mobile health platforms to provide regular guidance to patients, improve the standardization of patient self-care and the accuracy of self-assessment, and optimize the type of stoma device and accessory combination in a timely manner (54).

Additionally, accurately customizing the aperture of the skin barrier to accommodate dynamic morphological changes is crucial for minimizing peristomal complications. Reducing the inferiority and anxiety inextricably linked to such complications serves as an additional buffer against stigma. Regular and timely emptying of the ostomy pouch is also essential for maintaining stoma hygiene and preventing odor, thereby indirectly reducing experiences of discrimination by minimizing socially uncomfortable or embarrassing situations.

Regarding the optimal pouch-emptying threshold, UpToDate recommends emptying when the pouch reaches one-third of its total capacity, whereas the Chinese guideline suggests a range of one-third to one-half capacity (24, 26). While the 5S evidence pyramid model generally prioritizes evidence derived from UpToDate, this discrepancy likely reflects variations in care settings, patient characteristics, and product specifications. To maximize the feasibility and adherence of daily stoma management, this study recommends adopting the one-third to one-half capacity threshold. Ultimately, the synthesized evidence demonstrates that these routine stoma care measures are not only of paramount clinical importance but also furnish valuable indirect evidence for ameliorating stigma among patients with colorectal cancer and stomas.

### 4.5 Equal emphasis on self-management and social support is the cornerstone for alleviating the stigma of patients

The presence of a stoma frequently presents patients persistent challenges related to dietary habits, lifestyle, and daily activities. These challenges not only impairs patients’ ability to perform activities of daily living but also severely undermines their autonomy and self-efficacy, rendering them unable to promptly and effectively implement interventions when facing stoma-related issues during home care, thereby imposing a heavy burden on both the patients and their families (55, 56). Self-management ability plays a core regulatory role in this process. By enhancing the patients’ self-management ability, it can effectively strengthen their coping ability in the face of unexpected enterostomy situations, thereby reducing the experience of stigma-related negative emotions (57). Multiple studies have demonstrated that various forms of psychological interventions can improve patients’ confidence and capability in coping with stigma (34, 38–40). Therefore, evidence supporting self-management and psychological interventions may help reduce negative self-perception and improve self-acceptance, thereby alleviating internalized stigma.

However, the causes of stigma are not only related to individual factors but also closely linked to the internalization of individual social evaluations (8). Research indicates that the experience of stigmatization involves aberrant activation in brain regions associated with emotional processing and the perception of social threats, such as the amygdala, insula, anterior cingulate cortex, and hippocampus, accompanied by prefrontal cortical dysregulation. This distinct neural activity pattern can heighten an individual’s sensitivity to negative social cues and reinforce social avoidance behaviors, potentially exposing the patient to more severe psychosocial exclusion upon societal reintegration (13, 58). Therefore, merely relying on improving self-management ability to counter the overwhelming social psychological pressure may be insufficient. The dynamic interaction between self-management and social support has become a critical component of stigma intervention. On the one hand, the emotional support provided by family members and close social relationships may help patients rebuild their sense of identity and reintegrate into social activities. On the other hand, adequate social support systems can address both perceived and actual stigma by reshaping public attitudes and curtailing discriminatory behaviors (47, 59). Therefore, integrating multidimensional psychological interventions, maximizing family involvement, and cultivating robust social support networks can yield synergistic benefits. This approach not only disrupts the consolidation of negative cognitions tied to internalized stigma but also utilizes empathy-based support to rescue patients from “pathological isolation”, thereby neutralizing the detrimental impact of stigma on self-identity through the lens of social recognition (38–40).

Although evidence regarding psychosocial support is supported by multiple randomized controlled trials, all of these studies were conducted in China. Variations in nursing models, cultural backgrounds, and social values may limit the transferability of these findings to healthcare environments characterized by different social structures and support systems. Therefore, cultural adaptation should be thoroughly considered during evidence implementation and appropriate contextual modifications should be made to ensure the effective translation of this evidence into clinical practice.

### 4.6 Follow-up care that combines professionalism, continuity, and targeted approaches supports long-term intervention for stigma

Since most patients with colorectal cancer and stomas undergo home-based rehabilitation, the focus of their care extends from the hospital setting to community and home environments. Therefore, the role of follow-up in continuous care system has become increasingly important. Currently, the intervention measures for stigma in this population mainly focus on short-term effects, lacking systematic and continuous long-term follow-up plans, which to some extent weakens the continuity of care (45). However, given the intricate and enduring nature of stoma management, patients routinely confront a multitude of care-related challenges during the peri-discharge transition, which can inevitably trigger or exacerbate stigma (60). Therefore, initiating follow-up during the early postoperative phase and sustaining it throughout the outpatient recovery trajectory is critical for bridging the gap between inpatient and community care (31). Stoma specialist nurses not only possess comprehensive and systematic knowledge of stoma-related issues and the ability to address them but also can sensitively identify patients’ potential emotional changes and psychological needs. Having stoma specialist nurses lead the integration of mental health education into the follow-up process, enables the concurrent assessment and management of both physical complications and psychological distress (34, 35). An integrated “physical-psychological” follow-up model anchored by stoma specialist nurses can provide patients with sustained feedback, support, and positive reinforcement. Through targeted cognitive guidance, this model enhances patient self-efficacy and mitigates negative self-labeling, thereby curbing the development of stigma during long-term rehabilitation.

However, differences in factors such as the level of economic development, the characteristics of the medical environment and the ability of professionals, may limit the generalizability and feasibility of this integrated follow-up model in the application of some regions or countries with limited resources. Therefore, it is necessary to comprehensively evaluate the clinical practice and judiciously apply this evidence.

### 4.7 Limitations

Several limitations of this study should be acknowledged. First, this study included only Chinese and English literature, which may have resulted in the omission of relevant evidence published in other languages. Second, the synthesized evidence was derived from diverse study designs, and variations in research objectives, methodologies, and outcome measures reduced the comparability among studies, thereby affecting the consistency of the extracted evidence. Third, the interventions used in the included randomized controlled trials were relatively limited and failed to explore the effects of different interventions on stigma. Fourth, owing to a paucity of high-level evidence specifically targeting stigma, such as clinical decisions, clinical practice guidelines, and expert consensus, some indirect evidence had to be incorporated during the evidence synthesis process, which may have reduced the specificity of certain recommendations.

Therefore, future research should encompass multilingual literature while rigorously controlling for methodological heterogeneity. In addition, high-quality randomized controlled trials evaluating diverse interventions are warranted to generate high-level evidence specifically targeting stigma.

## 5 Conclusion

This study provides a systematic synthesis of the best available evidence for screening and managing stigma in patients with colorectal cancer and stomas, yielding actionable practice recommendations across seven core domains: team construction, stigma identification and assessment, educational guidance, stoma and appliance management, self-management, psychosocial support, and follow-up. The synthesized evidence provides a structured basis for clinical screening and management of stigma in patients with colorectal cancer and stomas.

During evidence implementation, clinical realities, individual patient differences, and family and social contexts should be fully considered, and appropriate evidence should be selected critically. Evidence related to stigma identification and assessment, self-management, stoma and appliance management, and psychosocial support demonstrated stronger relevance and broader coverage for stigma screening and management; therefore, these domains should be prioritized in clinical practice. Furthermore, future research should focus on developing stigma-specific guidelines and stigma assessment tools that are culturally appropriate, as well as conducting high-quality randomized controlled trials evaluating multiple interventions to further expand the evidence base.
