# Supplementary material for: Best evidence summary of screening and management of stigma in patients with colorectal cancer and stomas
Source: Front Oncol. 2026 Jun 4;16:1841569. doi: 10.3389/fonc.2026.1841569 (PMC13275223; doi:10.3389/fonc.2026.1841569)
Supplement: Supplementary file 1 [file Table1.docx]

PubMed (271)

| #1 | "Enterostomy"[Mesh] Sort by: Most Recent | 20573 |
| --- | --- | --- |
| #2 | ostomy[Title/Abstract] OR stoma[Title/Abstract] OR enterostomy[Title/Abstract] OR colostomy[Title/Abstract] OR ileostomy[Title/Abstract] | 30593 |
| #3 | "Social Stigma"[Mesh] Sort by: Most Recent | 17162 |
| #4 | social stigma[Title/Abstract] OR stigma[Title/Abstract] OR shame[Title/Abstract] OR embarrassment[Title/Abstract] OR guilt[Title/Abstract] OR discrimin*[Title/Abstract] OR humiliat*[Title/Abstract] OR dishonor[Title/Abstract] OR mortification[Title/Abstract] OR disgrace*[Title/Abstract] | 450912 |
| #5 | (#1 OR #2) AND (#3 OR #4) | 271 |

Cochrane Library (24)

| #1 | MeSH descriptor: [Enterostomy] explode all trees | 664 |
| --- | --- | --- |
| #2 | (ostomy OR stoma OR enterostomy OR colostomy OR ileostomy):ti,ab,kw | 3153 |
| #3 | #1 OR #2 | 3257 |
| #4 | MeSH descriptor: [Social Stigma] explode all trees | 884 |
| #5 | (social stigma):ti,ab,kw OR (stigma):ti,ab,kw OR (shame):ti,ab,kw OR (embarrassment):ti,ab,kw OR (guilt):ti,ab,kw OR (discrimin*):ti,ab,kw OR (humiliat*):ti,ab,kw OR (dishonor):ti,ab,kw OR (mortification):ti,ab,kw OR (disgrace*):ti,ab,kw | 20830 |
| #6 | #4 OR #5 | 20830 |
| #7 | #3 AND #6 | 24 |

Embase (505)

| #1 | 'enterostomy'/exp | 53029 |
| --- | --- | --- |
| #2 | ostomy:ti,ab,kw OR stoma:ti,ab,kw OR enterostomy:ti,ab,kw OR colostomy:ti,ab,kw OR ileostomy:ti,ab,kw | 50058 |
| #3 | #1 OR #2 | 76727 |
| #4 | 'stigma'/exp | 32158 |
| #5 | 'social stigma':ti,ab,kw OR stigma:ti,ab,kw OR shame:ti,ab,kw OR embarrassment:ti,ab,kw OR guilt:ti,ab,kw OR discrimin*:ti,ab,kw OR humiliat*:ti,ab,kw OR dishonor:ti,ab,kw OR mortification:ti,ab,kw OR disgrace*:ti,ab,kw | 585700 |
| #6 | #4 OR #5 | 591028 |
| #7 | #3 AND #6 | 505 |

Web of Science (750)

| #1 | ostomy OR stoma OR enterostomy OR colostomy OR ileostomy (Topic) | 43248 |
| --- | --- | --- |
| #2 | social stigma OR stigma OR shame OR embarrassment OR guilt OR discrimin* OR humiliat* OR dishonor OR mortification OR disgrace* (Topic) | 867781 |
| #3 | #1 AND #2 | 750 |

CINAHL (102)

| S1 | MH "Enterostomy" OR TI (ostomy OR stoma OR enterostomy OR colostomy OR ileostomy) OR AB (ostomy OR stoma OR enterostomy OR colostomy OR ileostomy) | 9374 |
| --- | --- | --- |
| S2 | MH "Stigma" OR TI (social stigma OR stigma OR shame OR embarrassment OR guilt OR discrimin* OR humiliat* OR dishonor OR mortification OR disgrace*) OR AB (social stigma OR stigma OR shame OR embarrassment OR guilt OR discrimin* OR humiliat* OR dishonor OR mortification OR disgrace*) | 115519 |
| S3 | S1 AND S2 | 102 |
